# Rheumatological Manifestations of Systemic Amyloidosis: A Retrospective Single-Centre Study From a Tertiary Care Hospital in India

**DOI:** 10.7759/cureus.105451

**Published:** 2026-03-18

**Authors:** Manikandan Gopal, Abraham George N, Navaneeth Kamath, Koshy Nithin Thomas, Ruchika Goel, John Mathew

**Affiliations:** 1 Department of Clinical Immunology and Rheumatology, Christian Medical College Vellore, Vellore, IND

**Keywords:** aa amyloidosis, al amyloidosis, axial spondyloarthritis, cardiac amyloidosis with reduced ejection fraction, inflammatory arthritis of the small joints, localized amyloid, nephrotic range proteinuria, renal aa amyloidosis, rheumatoid arthritis, secondary systemic amyloidosis

## Abstract

Objective: Systemic amyloidosis may present with rheumatological features that mimic inflammatory arthritis, frequently leading to significant diagnostic delays. This study describes the clinical profile, patterns of organ involvement, and clinical outcomes of patients with systemic amyloidosis who presented to a rheumatology clinic.

Methods: A retrospective observational study with review of electronic medical records from January 2010 to December 2024 at Christian Medical College, Vellore, identified patients diagnosed with amyloidosis who presented with specific rheumatological complaints. Data on clinical features, organ involvement, biopsy findings, amyloid typing, treatment, and survival were analyzed.

Results: In this retrospective observational study spanning January 2010 to December 2024 and including 24 patients with systemic amyloidosis, amyloid A (AA) amyloidosis was the most prevalent type (n=11, 45.8%), followed by amyloid light-chain (AL) amyloidosis (n=seven, 29.2%), with the remaining six cases (25.0%) classified as amyloidosis of unspecified type. Of 24 patients, the most common presenting features were peripheral joint pain (15 patients, 62.5%), early morning stiffness (15 patients, 62.5%), and axial joint pain (nine patients, 37.5%). Classic signs included macroglossia (four patients, 16.6%), carpal tunnel syndrome (three patients, 12.5%), and periorbital purpura (three patients, 12.5%). Renal involvement with proteinuria exceeding 1 gram in 24 hours was seen in 12 patients (50%), and chronic kidney disease was noted in five patients (20.8%). Cardiac amyloidosis was confirmed in seven patients (29%). Biopsy showed vessel wall deposition in all cases (24 patients, 100%). Amyloid typing revealed AL in seven patients, AA in 11, and unspecified types in six. AA amyloidosis showed significantly better survival (90.9%, one death in 11 patients) than AL amyloidosis (14.3%, six deaths in seven patients), yielding a highly significant difference (p = 0.0033). This confirms markedly poorer survival in the AL group compared to the AA group. Cardiac involvement was associated with markedly poorer prognosis, accounting for six of the seven total deaths (p = 0.0003).

Conclusion: Systemic amyloidosis must remain a key differential diagnosis for any patient presenting with atypical inflammatory arthritis accompanied by early morning stiffness, especially when features such as soft tissue expansion or cutaneous bleeding are present. A high index of clinical suspicion is essential, particularly when joint symptoms are accompanied by specific red-flag signs such as macroglossia, carpal tunnel syndrome, or periorbital purpura. Cardiac involvement portends poor prognosis, particularly in the AL type.

## Introduction

Systemic amyloidosis is a rare disorder characterized by extracellular deposition of misfolded proteins, leading to organ dysfunction. Epidemiological data remain limited, largely due to the disease's low incidence and the absence of a global network of population-based registries specific to this condition [1]. It can be classified as primary (amyloid light-chain (AL)), secondary (amyloid A (AA), associated with chronic inflammation), or other types [2]. Rheumatological manifestations are common, particularly in AL amyloidosis, where arthropathy, morning stiffness, and polyarthritis may mimic rheumatoid arthritis [3]. Secondary AA amyloidosis is a known complication of longstanding inflammatory arthritis, such as rheumatoid arthritis and spondyloarthritis [4]. However, primary amyloidosis presenting primarily to rheumatologists is underrecognized, potentially delaying diagnosis and worsening prognosis, especially with cardiac involvement [5].

This retrospective study from a single tertiary center in India describes the rheumatological presentation, organ involvement patterns, amyloid typing, and outcomes in patients with amyloidosis evaluated in a rheumatology clinic over 14 years.

## Materials and methods

This was a retrospective observational study conducted at the Department of Clinical Immunology and Rheumatology, Christian Medical College, Vellore, a tertiary referral hospital in South India. Electronic medical records (EMRs) from January 2010 to December 2024 were searched for the diagnosis of "Amyloidosis" in patients presenting with specific rheumatological complaints (e.g., joint pain, stiffness, or arthritis). Patients with confirmed amyloidosis on biopsy and systemic involvement were included. The study excluded patients based on the following criteria: pre-existing diabetic nephropathy or hypertensive nephrosclerosis that would confound the assessment of renal amyloidosis or proteinuria exceeding one gram in 24 hours; a lack of a definitive tissue biopsy confirming amyloid deposition; incomplete medical records regarding amyloid typing; age under 18 years; pregnant female; the presence of severe valvular heart disease or ischemic cardiomyopathy determined to be clearly unrelated to amyloid deposition.

Data collected included demographics, clinical features, laboratory findings, echocardiographic and cardiac MRI results, biopsy sites and findings, amyloid typing (AL, AA, or unspecified), underlying conditions, treatment, and survival status. The principal investigator reviewed each of these records. Exclusions were applied sequentially during EMR screening and patients meeting exclusion criteria were removed. Diagnosis of amyloidosis was based on Congo red-positive deposits with apple-green birefringence, and typing where available. Cardiac involvement was defined by echocardiographic speckling, thickened myocardium, diastolic dysfunction, or confirmatory cardiac MRI/biopsy. Institutional ethics committee approval was obtained. Patient consent was waived due to the retrospective nature.

Data was entered into an Excel sheet (Microsoft Corp., Redmond, WA, USA) and analyzed. Mean values were calculated for approximately normally distributed data, and the median was calculated for skewed data. Because of the small sample size, a Fisher’s exact test was used to determine if the difference in mortality is statistically significant.

## Results

The study cohort comprised 24 patients, with an equal distribution of 12 male and 12 female participants. The median age of the study population was 45.5 years (IQR 41.5-53.75). For hospitalized patients, the mean duration of stay prior to a definitive diagnosis was 11.7 ± 2.9 days. The duration of symptoms before diagnosis exhibited a wide variation, ranging from one to 288 months. Regarding amyloid classification, AA amyloidosis was the most prevalent type, accounting for 11 cases, followed by AL amyloidosis in seven cases. The remaining six cases were classified as amyloidosis of an unspecified type.

Causes of AA amyloidosis

**Figure 1:**
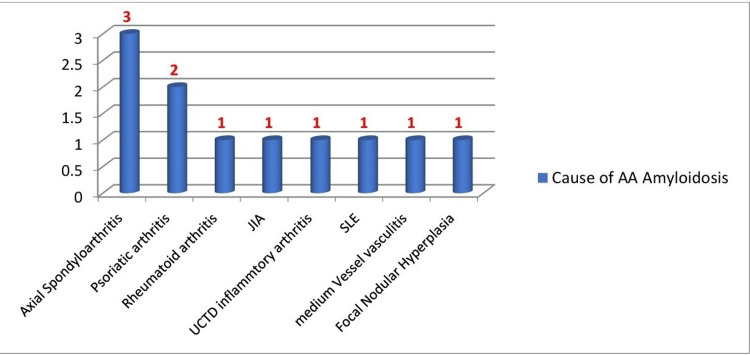
Causes of secondary (AA) amyloidosis (n = 11) x axis - Causes of AA amyloidosis y axis - Number of patients AA: amyloid A, JIA: juvenile idiopathic arthritis, SLE: systemic lupus erythematosus, UTCD: undifferentiated connective tissue disease

In our patient cohort, the primary rheumatological diagnoses associated with a chronic inflammatory burden that led to the development of AA amyloidosis included axial spondyloarthritis, psoriatic arthritis, rheumatoid arthritis, undifferentiated connective tissue disease, systemic lupus erythematosus, and medium-vessel vasculitis. Additionally, one patient in the cohort had a non-rheumatological diagnosis of focal nodular hyperplasia of the liver (Figure 1).

**Figure 2:**
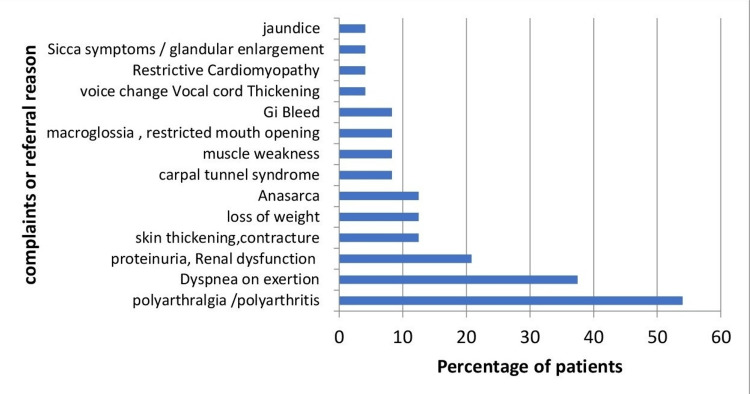
Presenting complaints or reasons for referral to the Rheumatology department among 24 patients with amyloidosis x axis - Percentage of patients y axis - Presenting complaint or referral reasons

The common symptoms, signs, or reasons for referral at the baseline visit to the rheumatology clinic included polyarthralgia, skin thickening, weight loss, muscle weakness, sicca symptoms, melena, unexplained proteinuria or renal dysfunction, anasarca, carpal tunnel syndrome, macroglossia, and thickening of the vocal cords with voice change (Figure 2).

Clinical features

The clinical features of the patients were retrospectively analyzed from the time of symptom onset following a definitive diagnosis of amyloidosis. The most prevalent symptoms were peripheral joint pain and early morning stiffness, each affecting 15 patients (62.5%). These were followed by axial joint pain, which occurred in nine patients (37.5%). Less frequent but clinically significant features included macroglossia in four patients (16.6%), as well as carpal tunnel syndrome and periorbital purpura, each present in three (12.5%) of the patients studied (Table 1).

**Table 1:** Clinical features observed in 24 patients diagnosed with amyloidosis

S No	Clinical Features	N=24 (%)
1	Peripheral joint pain	15 (62.5)
2	Early morning stiffness	15 (62.5)
3	Axial joint pain	9 (37.5)
4	Macroglossia	4 (16.6)
5	Carpal Tunnel Syndrome	3 (12.5)
6	Periorbital purpura	3 (12.5)

Organ involvement

At the time of diagnosis, renal involvement with proteinuria exceeding 1 gram per day was present in 12 patients (50%), with mean serum creatinine 1.21 ± 0.37 mg/dL. Following one year of follow-up, chronic kidney disease had developed in five patients (20.8%). Cardiac amyloidosis was screened for and confirmed using echocardiography, cardiac magnetic resonance imaging (MRI), and myocardial biopsy when feasible. Echocardiographic abnormalities included thickened left ventricle (LV) and right ventricle (RV) walls, myocardial speckling, thickened interatrial septum, reduced tissue velocities, relative apical sparing on strain imaging, diastolic dysfunction, systolic dysfunction, global hypokinesia, and low cardiac output. Cardiac MRI abnormalities observed in our patients included myocardial wall thickening, reversal of the nulling pattern, and subendocardial late gadolinium enhancement. Cardiac amyloidosis was confirmed in seven patients (29%), based on myocardial speckling on echocardiography in six patients, cardiac MRI findings in two patients, and biopsy demonstrating amyloid deposition in two patients. This diagnostic approach is consistent with the European Society of Cardiology (ESC) Working Group on Myocardial and Pericardial Diseases position statement on the diagnosis and treatment of cardiac amyloidosis.

The most frequent biopsy site was the kidney, followed by several alternative sites including the abdominal fat pad, stomach, rectum, myocardium, labial salivary gland (lip biopsy), and vocal cord. Notably, vessel wall amyloid deposition was identified in all biopsy specimens, occurring in 100% of the samples analyzed.

Underlying conditions and management

The primary rheumatological diagnoses - including axial spondyloarthritis, psoriatic arthritis, rheumatoid arthritis, undifferentiated connective tissue disease, systemic lupus erythematosus, and medium-vessel vasculitis - were managed using standard treatment regimens. These included corticosteroids, conventional synthetic disease-modifying antirheumatic drugs (DMARDs) such as methotrexate and sulfasalazine, as well as cyclophosphamide, rituximab, and biologics where clinically indicated. Patients diagnosed with AL amyloidosis received chemotherapy regimens, and one patient underwent autologous bone marrow transplantation. Notably, none of the patients in this cohort received amyloid-specific therapies.

Outcomes and prognosis

Regarding follow-up, four patients were lost to further evaluation. Among the remaining patients with available one-year follow-up data, seven deaths were recorded. AA amyloidosis showed significantly better survival (90.9%, one death in 11 patients) than AL amyloidosis (14.3%, six deaths in seven patients), yielding a highly significant difference (p = 0.0033). This confirms markedly poorer survival in the AL group compared to the AA group. Cardiac involvement was associated with markedly poorer prognosis, accounting for six of the seven total deaths (p = 0.0003). One patient without cardiac involvement died due to sepsis.

## Discussion

The demographic profile of our cohort reveals equal gender distribution and a predominantly middle-aged population, with a median age of 45.5 years. The wide variation in symptom duration before amyloidosis diagnosis (ranging from one to 288 months) emphasizes the considerable and sustained inflammatory burden responsible for AA amyloidosis, whereas AL amyloidosis usually develops more rapidly. This variability also illustrates the diagnostic challenges commonly faced in rheumatological practice, where non-specific initial symptoms often lead to considerable delays in referral to specialized care [6]. Furthermore, the mean hospital stay of approximately 11.7 ± 2.9 days in a tertiary care center suggests a high level of disease complexity or the need for intensive inpatient evaluation and stabilization [7].

In our cohort, joint pain was reported in 62.5% of patients, highlighting its potential as a prominent presenting or associated symptom in systemic amyloidosis. The high prevalence observed here underscores the need for rheumatologists and clinicians to maintain a low threshold for considering amyloidosis in patients with persistent, unexplained arthralgias or inflammatory features, especially when accompanied by systemic signs such as proteinuria or organ dysfunction [8]. Unlike traditional inflammatory arthritis, amyloid arthropathy results from the direct infiltration of amyloid fibrils into the synovium and periarticular structures [9]. This "non-inflammatory" infiltration can still present with significant morning stiffness, leading clinicians to rely on laboratory markers like rheumatoid factor (RF) and anti-cyclic citrullinated peptide (anti-CCP), which were negative in the majority of our atypical cases [10]. Early recognition could prompt targeted evaluation (e.g., serum amyloid A levels, biopsy) and control of the underlying inflammation to prevent progression to irreversible organ damage. Classic signs like macroglossia, carpal tunnel, and periorbital purpura were less frequent but diagnostic when present [11]. Macroglossia, found in 16.6% of our patients, is highly suggestive for AL type when seen in an adult presenting with joint pain [12]. Similarly, periorbital ooze (or "raccoon eyes") indicates capillary fragility due to amyloid deposition in the vessel walls. These findings are rarely seen in secondary (AA) amyloidosis [13]. Renal involvement was common (50%), consistent with the literature. Similarly, cardiac involvement was identified in 29% of patients and was strongly linked to adverse outcomes, accounting for the majority (six out of seven) of deaths (p = 0.0003) [14,15]. Our data emphasize the importance of routine cardiac screening (e.g., echocardiography with strain imaging, cardiac MRI) in all amyloidosis patients, regardless of subtype, to enable early detection, multidisciplinary management (e.g., heart failure optimization, arrhythmia monitoring), and improved risk stratification.

The transition from clinical suspicion to histological confirmation of AA or AL is vital. This distinction is therapeutically paramount: AA amyloidosis requires aggressive control of the underlying inflammatory disease (e.g., TNF inhibitors for axial spondyloarthritis), whereas AL amyloidosis requires hematological intervention to address the underlying plasma cell dyscrasia [16]. AL amyloidosis showed worse outcomes compared to AA, aligning with reports that cardiac involvement in AL confers a median survival of months if untreated [17]. In contrast, AA patients had better survival, likely due to control of underlying inflammation [18-20].

Limitations

Our study has several limitations that should be considered when interpreting the results. Primarily, the retrospective nature of the data collection from EMRs may have introduced inherent biases or resulted in incomplete data points. Referral bias inherent to a tertiary rheumatology center and follow-up data were unavailable for a few patients who were lost to follow-up, limiting our ability to draw reliable conclusions regarding treatment response. Additionally, the small sample size of 24 patients from a single tertiary care center may restrict the generalizability of our findings to broader populations.

Despite these limitations, the study possesses notable strengths, such as a detailed multi-organ assessment conducted specifically within a rheumatology clinical setting. The long study duration (14 years) is a strength but may also introduce heterogeneity in diagnostic and therapeutic approaches over time.

## Conclusions

Joint pain was reported in 62.5% of patients, highlighting its potential as a prominent presenting or associated symptom in systemic amyloidosis. It should therefore remain a key differential diagnosis in patients presenting with atypical inflammatory arthritis accompanied by early morning stiffness - particularly when features such as soft tissue expansion or cutaneous bleeding are present. A high index of clinical suspicion is essential, especially when joint symptoms coexist with red-flag signs such as macroglossia, carpal tunnel syndrome, or periorbital purpura. Although these classic features may be less frequent, they are highly specific, particularly for AL amyloidosis, and should prompt immediate further investigation in any adult presenting with unexplained joint pain.

Early recognition, followed by biopsy confirmation and accurate distinction between AL and AA types, enables tailored therapy: suppression of underlying inflammation in AA amyloidosis and plasma cell-directed treatment in AL amyloidosis. This approach can potentially halt organ damage and improve survival, particularly if instituted before advanced cardiac involvement develops. Cardiac involvement was identified in 29% of patients in our study and remains the major determinant of poor prognosis, especially in the AL type. Ultimately, prompt recognition of these atypical features and multi-organ involvement is essential to initiate organ-saving therapy and enhance overall patient survival. Larger multicenter studies are required to confirm the trends observed in this study.
